# IL6-receptor antibody tocilizumab as salvage therapy in severe chronic graft-versus-host disease after allogeneic hematopoietic stem cell transplantation: a retrospective analysis

**DOI:** 10.1007/s00277-020-03968-w

**Published:** 2020-02-21

**Authors:** Anna-Sophia Kattner, Ernst Holler, Barbara Holler, Sebastian Klobuch, Daniela Weber, Danilo Martinovic, Matthias Edinger, Wolfgang Herr, Daniel Wolff

**Affiliations:** Department of Internal Medicine III, Hematology & Internal Oncology, University Hospital Regensburg, Franz-Josef-Strauss-Allee 11, 93053 Regensburg, Germany

**Keywords:** Allogeneic hematopoietic stem cell transplantation, Chronic graft-versus-host disease, Tocilizumab, Interleukin-6

## Abstract

Chronic graft-versus-host disease (cGvHD) remains the most relevant factor affecting survival after allogeneic hematopoietic stem cell transplantation (alloHSCT). Besides corticosteroids (and ibrutinib in the USA), there is no established therapy for cGvHD. Tocilizumab, a humanized IgG1 IL6-receptor antibody, has shown efficacy in acute GvHD and cGvHD. We retrospectively analyzed the efficacy and safety of tocilizumab for the treatment of advanced cGvHD. Eleven patients with severe steroid refractory cGvHD (median age 49; range 21–62 years) that received at least two prior lines of therapy for cGvHD (range 2–8 regimens) were treated with tocilizumab (q4w, dosage 8 mg/kg IV) with a median number of 15 cycles (range 2–31). NIH consensus criteria grading for cGvHD were recorded prior to tocilizumab administration and after 3, 6, and 12 months of therapy. All patients received additional concomitant immunosuppression (IS) but no new IS within the last 4 weeks before start of tocilizumab and response assessment was terminated before start of any new IS. The median number of days between alloHSCT and initiation of tocilizumab therapy was 1033 days. Organs involved at initiation of tocilizumab therapy were skin (100%, all grade 3), eyes (82%), fascia (82%), mouth (64%), lungs (55%), and genitals (18%). Overall, 7/10 patients (70%) showed partial remission, 2/10 patients (20%) showed progressive cGvHD, 1/10 patient (10%) showed mixed response, and 1 patient died due to sepsis before first response assessment 1.5 months after initiation of treatment. Four patients required subsequent new immunosuppressive treatment. Two patients developed bacterial sepsis, one of whom died. The overall survival and relapse-free survival were 82% with an average follow-up of 22 months (range 1.5–52 months). Tocilizumab seems a promising treatment option in advanced cGvHD but further evaluation within a phase II trial is required.

## Introduction

Chronic graft-versus-host disease (cGvHD) after allogeneic hematopoietic stem cell transplantation (alloHSCT) remains the most significant long-term complication associated with increased morbidity and mortality as well as reduced quality of life [1]. Established first-line therapy for cGvHD still consists of corticosteroids and calcineurin inhibitors [2]. However, about half of the patients with cGvHD are refractory to therapy with corticosteroids, and there are no approved drugs for these patients except the newly FDA-approved B cell–targeting agent ibrutinib [3]. For various experimental or compassionate use treatments, response rates usually range from 30 to 60% [3, 4]. Therefore, it is essential to identify other substances for adequate therapy of steroid refractory cGvHD.

The cytokine interleukin 6 (IL-6) plays a significant role in the initiation of the inflammatory response in different situations including tissue damage, infection, autoimmune disease as well as GvHD through activation of the signal transducer and activator of transcription 3 (STAT3) and mitogen activated protein (MAP) kinase pathways [5, 6]. The activation of these pathways leads to increased immunoglobulin production by B cells, increased plasmablast count, and decreased differentiation of regulatory T cells (Tregs). In addition, IL-6 induces differentiation of (especially IL-17 producing) T helper cells known to be involved in inflammatory states [7].

Tocilizumab is a humanized IgG1 IL-6 receptor antibody approved by the EMA for the first time in 2009. Meanwhile, the drug is approved for the treatment of rheumatoid arthritis [8], active systemic juvenile idiopathic arthritis [9], giant cell arteritis [10], and cytokine release syndrome following CAR T cell infusion [11], all of which are known to be IL-6 mediated diseases. Therefore, tocilizumab seems a promising substance for treatment of steroid refractory cGvHD which often resembles inflammatory autoimmune disease.

Previous studies have shown efficacy of soluble IL-6 receptor (sIL-6R) blockade with tocilizumab for the prevention of acute GvHD in murine models and early clinical trials by the inhibition of the abovementioned signaling pathways in monocytes and mature donor T cells (without altering graft-versus-leukemia effects) as well as through the inhibition of direct toxic effects of IL-6 [6, 12]. In murine models, IL-6 promotes donor B cell–derived IgG-induced Th17 cell infiltration and germinal center formation and thereby contributes to GVHD pathogenesis [13, 14], as also seen in solid organ transplant models [15, 16]. However, there are few studies describing the effects of tocilizumab on the development and treatment of cGvHD in patients after alloHSCT [17, 18]. We retrospectively analyzed the efficacy and safety of tocilizumab for the treatment of advanced cGvHD at our center between the years 2015 and 2019.

## Patients and methods

### Patients

All patients at our transplant center treated with tocilizumab for advanced steroid refractory cGvHD were included in a retrospective analysis (*n* = 11). Diagnosis and response assessment were performed according to the criteria of the National Institute of Health (NIH) consensus [19]. Patients received monthly infusions of tocilizumab at a dosage of 8 mg/kg with a maximum dose of 800 mg over the period of 1 h for at least two cycles. The retrospective analysis was conducted by chart review. All procedures followed were in accordance with the ethical standards of the responsible committee on human experimentation (institutional [approval no. 19-1482-104] and national) and with the Helsinki Declaration of 1975 as revised in 2008. Informed consent was obtained from all patients for being included in the study.

### Definition of tocilizumab response

Response was assessed at 3, 6, and 12 months after treatment with tocilizumab or at the time of progression of cGvHD and onset of a new therapy during routine follow-up visits. Last day of follow-up was September 2, 2019.

Complete remission (CR) was defined as resolution of all organ manifestations and symptoms of cGvHD, partial remission (PR) as improvement by at least one organ grade without progression at other sites, stable disease (SD) if no change occurred, and progressive disease (PD) if worsening in at least one organ occurred [20]. All patients received additional concomitant immunosuppressive agents, but no new treatment modality for cGvHD within the last 4 weeks before start of tocilizumab and last response assessment was performed before the start of any additional new IS.

## Results

### Patient characteristics

Since the year 2015, 11 patients were treated with tocilizumab for advanced cGvHD (see Table 1 for details). All patients had received peripheral blood stem cells as stem cell source, and all but one patient received HLA-matched grafts. Seven of the eleven donors were unrelated (64%) and four were related (36%). GvHD prophylaxis consisted of a calcineurin inhibitor in combination with methotrexate or mycophenolate, and in case of unrelated donors, ATG was added to the conditioning regimen. One patient received everolimus as GvHD prophylaxis (3rd alloHSCT for advanced AML). The median number of days between alloHSCT and onset of cGvHD was 202 (range 89–545) and 9/11 patients showed quiescent cGvHD (81%) including one patient with initial overlap syndrome, and 2/11 patients developed de novo cGvHD (18%). At cGvHD onset, 8/11 patients had mild cGvHD and 3/11 patients had moderate cGvHD. The platelet count at onset of cGvHD was < 100/nl in 5/11 patients. Organs involved at initiation of tocilizumab therapy were skin (100%, all grade 3), eyes (82%), fascia (82%), mouth (64%), lungs (55%), and genitals (18%), and the median number of therapy lines prior to start of treatment with tocilizumab was 4 (range 2–8). Prior therapy included prednisolone, everolimus, tacrolimus, rituximab, abatacept, ruxolitinib, bortezomib, total nodal irradiation (TNI), extracorporeal photopheresis (ECP), mycophenolate (MMF), cyclosporin, infusion of in vitro expanded regulatory Treg cells (Treg), ibrutinib, and cyclophosphamide (see Table 2). Tocilizumab therapy was initiated after a median of 1033 days after alloHSCT (range 482–1749); patients had a median age of 49 years (range 21–62). At the time of initiation of tocilizumab treatment, all patients had severe cGvHD including skin involvement with deep sclerosis. All patients received additional immunosuppressive agents in combination with tocilizumab but no new agent was started during the last 4 weeks before tocilizumab therapy, and response assessment was terminated at the time of initiation of a new immunosuppressive agent. Concomitant immunosuppressive agents included prednisolone (10/10 patients), ruxolitinib (6/10 patients), bortezomib (1/10 patients), ECP (1/10 patients), ibrutinib (1/10 patients), and everolimus (1/10 patients) (see Table 3). Patient characteristics are shown in Tables 1 and 2.

**Table 1:** Patient characteristics

Patient #	Age	Diagnosis–stage	Conditioning regimen	Donor type/gender match	GvHD prophylaxis
1	58	PMF–acceleration	FBM	MUD m to m	ATG, CyA, MMF
2	50	AML–1st CR	treo/flud	MUD m to m	ATG, CyA, MTX
3	21	AML–3rd PR	thio/flud	MMUD m to m	everolimus
4	62	CLL–Richter’s transformation	FBM	MUD f to m	ATG, CyA, MTX
5	41	AML–2nd CR	FBM	MRD f to f	CyA, MTX
6	44	AML–1st CR	FBM	MUD m to f	ATG, CyA, MTX
7	54	sAML–1st CR	FTM	MRD m to f	CyA, MTX
8	44	AML–1st CR	FTM	MUD m to f	ATG, tacrolimus, MMF
9	51	FL & CTLC–3rd PR	FTM	MRD f to f	CyA, MTX
10	40	ALL, Ph+–CR	flud/8 Gy TBI	MRD m to m	CyA, MTX
11	49	MM–SD	thio/treo/flud	MUD f to f	tacrolimus, MTX
Median	49				

*PMF*, primary myelofibrosis; *(s)AML*, (secondary) acute myeloid leukemia; *CLL*, chronic lymphocytic leukemia; *FL*, follicular lymphoma; *CTLC*, cutaneous T cell lymphoma; *ALL*, acute lymphoblastic leukemia; *MM*, multiple myeloma; *FBM*, fludarabine/busulfan/melphalan; *FTM*, fludarabine/thiotepa/melphalan; *treo*, treosulfan; *flud*, fludarabine; *thio*, thiotepa; *TBI*, total body irradiation; *MRD*, matched related donor; *MUD*, matched unrelated donor; *MMUD*, mismatch unrelated donor; *m*, male; *f*, female; *ATG*, anti-thymocyte globulin; *CyA*, cyclosporine; *MMF*, mycophenolate; *MTX*, methotrexate

**Table 2:** cGvHD characteristics

Patient #	Age	Day of onset cGvHD	Max aGvHD grade	cGvHD onset type	cGvHD grade at onset	Prior therapy for cGvHD	
1	58	215	3	qe	e1, lu1, m1	CS, everolimus, rituximab	
2	50	545	2	qe	s1	CS, everolimus, rituximab, bortezomib, ruxolitinib, TNRT	
3	21	202	3	qe	s2, m2, lu1	CS, everolimus, rituximab	
4	62	377	1	qe	s2, e1	CS, everolimus, ECP, ruxolitinib	
5	41	142	2	ov	m1	CS, CyA, MMF, everolimus, ECP, ruxolitinib, abatacept, Treg	
6	44	224	3	qe	s2, li1	CS, tacrolimus, MMF, rituximab, ruxolitinib, ibrutinib, abatacept, Treg	
7	54	155	2	qe	e3	CS, everolimus, ruxolitinib	
8	44	89	1	qe	m1	CS, everolimus	
9	51	222	0	no	m1	CS, everolimus, tacrolimus, MMF, ruxolitinib, Treg, ibrutinib	
10	40	198	0	no	f2	CS, everolimus, ibrutinib, ruxolitinib	
11	49	196	3	qe	e1	CS, MMF, cyclophosphamide, ruxolitinib, bortezomib	
Median	49	202	2				

*PC*, platelet count; *CS*, corticosteroids; *qe*, quiescent; *ov*, overlap; *no*, de novo; *e*, eyes; *lu*, lungs; *m*, mouth; *s*, skin; *li*, liver; *f*, fascia; *TNRT*, total nodal radiotherapy; *Treg*, regulatory T cell transfusion; *ECP*, extracorporeal photopheresis

**Table 3:** Tocilizumab data

Patient #	Day of start	No. of cycles	Organ involvement at start	3 m RR	6 m RR	12 m RR	Concomitant IS (delta t)	Follow-up (f/u) information	F/u in months
1	774	3	s3, e2, m1, f2, lu1	PD (f3)	-	-	CS (12m)	New IS with ruxolitinib, bortezomib, Tregs	50
2	854	31	s3, e1, f3	SD	SD	PR (f2)	CS (8m), ruxolitinib (4m), bortezomib (1.5m), ECP (− 2m–5m)	Reduction of IS: bortezomib, ruxolitinib, ECP d/c	31
3	617	4	s3, m1, e2, ge2, f1	PR (e0)	-	-	CS (14m)	new IS: ruxolitinib after PD (fascia), death due to relapse 4y after tocilizumab	52
4	1309	14	s3, m1, lu1, f1	PD (e1, f2)	-	-	CS (30m), ibrutinib (6m)	PD during new IS with Treg, restart of tocilizumab after 4m	14
5	1749	2	s3, m1, e1, lu1, f1	-	-	-		Death due to sepsis 6w after tocilizumab start, response n.a.	1.5
6	1033	15	s3, e3, f2, lu2	PR (e1)	PR (s2, f1)	PR (lu1)	CS (> 26m), ruxolitinib (5m)	Hospitalization due to sepsis, improvement of skin, ruxolitinib d/c	12
7	482	19	s3, e2	MR (s1, lu1)	MR (lu0, m1)	PD (s3, lu1)	CS (> 8m), ruxolitinib (7m)	PD after reduction of IS & 6w tocilizumab intervals	25
8	1309	16	s3, m2, e1, lu1, ge2	SD	SD	PR (m1, ge1)	CS (> 25m), everolimus (25m)	Improvement of oral and genital cGvHD	12
9	1245	6	s3, m2, ge2, f3, lu1	PR (m1, f1)	-	-	CS (> 25m), ruxolitinib (23m)	New IS with imatinib at 3 m f/u	14
10	602	15	s3, e1, f2	PR (e0)	PR (f0)	PR (s2)	CS (13m), ruxolitinib (7m)	Significant improvement in all organ grades	12
11	1510	19	s3, m1, e2, f2	PR (f1)	SD	PR (s2, f1)	CS (> 35m), ruxolitinib (29m)	Improvement of skin and fascial cGvHD	16
Median	1033	15							14

cGvHD organ grades remained the same over time unless noted in parentheses

*3m/6m/12m RR*, 3-, 6-, and 12-month response rate, respectively; *s*, skin; *e*, eyes; *m*, mouth; *f*, fascia; *lu*, lungs; *ge*, genital; *PD*, progressive disease; *SD*, stable disease; *PR*, partial response; *MR*, mixed response; *Treg*, regulatory T cells; *IS*, immunosuppression; *d/c*, discontinued; *CS*, corticosteroids; *ECP*, extracorporeal photopheresis; *y*, years; *m*, months; *w*, weeks; *delta t*, time concomitant IS was given before initiation of treatment with tocilizumab

### Response to tocilizumab after 3 months

At 3-month follow-up after initiation of tocilizumab therapy, 5/10 patients (50%) showed partial remission, 2/10 patients (20%) showed stable disease, 2/10 patients (20%) showed progressive cGvHD, and 1/10 patient (10%) showed mixed response. One patient died due to pseudomonas sepsis after soft tissue infection of cGvHD-associated skin ulcers 6 weeks after initiation of tocilizumab precluding response assessment. Two patients progressing with cGvHD started with new immunosuppressive regimens, one shortly before and the other shortly after 3-month follow-up. Due to critical general conditions (with severe pulmonary impairment caused by sclerosis of the fascia of the chest leading to lung restriction), patient number 2 (see Table 3) received new additional immunosuppression with ECP 2 months after tocilizumab therapy. However, ECP was discontinued before 6-month follow-up since symptoms improved on combined ECP and tocilizumab therapy. This patient formally remained in SD during 3- and 6-month follow-up but achieved a PR at 12-months. We conclude that improvement was due to tocilizumab rather than ECP (which was stopped more than 6 months before PR was achieved) and therefore included the patient in the further analysis. The overall survival (OS) at 3-month follow-up was thus 91%, and the failure-free survival (FFS), defined as unnecessity to start additional new systemic immunosuppressive therapy and the absence of relapse- and non-relapse-related mortality, was 64% [21].

### Response to tocilizumab after 6 and 12 months

At 6-month follow-up, 4/10 (including the two already mentioned) patients had received other immunosuppressive agents and were thus excluded from response assessment. One of these patients returned to tocilizumab therapy 4 months after discontinuing tocilizumab due to rapid progression of cGvHD upon treatment with an alternative regimen and showed subsequently stable disease. Of the remaining patients, 2/10 developed a partial response (20%), 3/10 showed stable disease (30%), and 1/10 had a mixed response (10%). In these patients, the average prednisolone dose could be reduced from 0.16 mg/kg/day (range 0–0.42 mg/kg/day) at 3-month follow-up to 0.08 mg/kg/day (range 0–0.17 mg/kg/day) at 6-month follow-up. Failure-free survival was 55%.

At 12-month follow-up, five patients (50%) showed a partial response which developed from stable disease at 6-month follow-up in three patients. The other two patients had a prior partial response at 6 months that was maintained. One patient developed progressive disease after a mixed response at 6-month follow-up. This patient received new IS treatment after 12-month follow-up. The average prednisolone dose remained stable at 0.08 mg/kg/day (range 0–0.23 mg/kg/day) due to the patient with mixed response who received an escalated dose after concomitant immunosuppression with ruxolitinib was discontinued, and intervals between tocilizumab applications were increased. All other patients received equal or lower corticosteroid doses (average prednisolone dose in responders 0.06 mg/kg/day, range 0–0.09 mg/kg/day). Failure-free survival remained stable at 55%.

### Toxicity

Infectious complications were assessed according to WHO standards with the following categories: “hospital admission,” “admittance to intensive care unit,” and “death.” Two patients developed serious infectious complications requiring hospital admission. One patient developed respiratory infection with parainfluenza type 3 as well as port infection with *Staphylococcus epidermidis* requiring port explantation and IV antibiotic treatment. Infection was developed 3 months after initiation of tocilizumab treatment. This patient fully recovered and showed a good response to tocilizumab therapy achieving a PR at the 3-, 6-, and 12-month follow-ups.

Another patient developed lethal systemic blood stream infection with pseudomonas due to soft tissue infection of skin ulcers associated with sclerotic cGvHD 6 weeks after initiation of tocilizumab therapy. Of note, both patients with infectious complications developed granulocytopenia (a known common side effect of tocilizumab) treated with granulocyte colony–stimulating factor (GCSF) and thrombocytopenia during the infectious complication. All but one patient required immunoglobulin substitution during treatment with tocilizumab, which was already started before initiation of tocilizumab treatment. The remaining patient received immunoglobulin substitution until 1 year prior to initiation of tocilizumab therapy and did not require restart of substitution on tocilizumab. No additional toxicities were observed.

In the meantime, one patient died due to late rapid progressive relapse of AML 4 years after the last cycle of tocilizumab. Therefore, we do not assume relapse being associated with tocilizumab treatment.

## Discussion

**Fig. 1:**
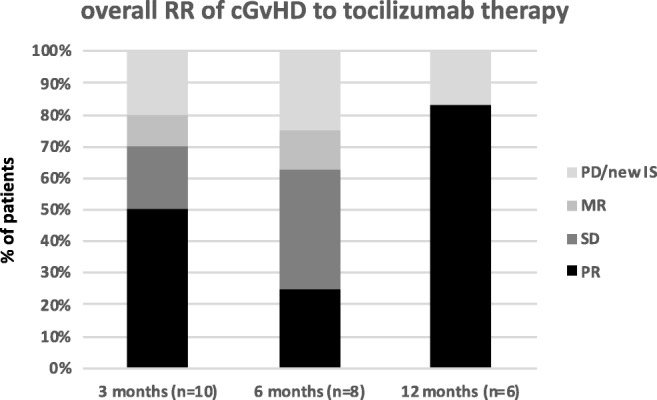
Overall response rates (RR). Overall response rates (RR) over time after initiation of tocilizumab therapy: RR are shown in percentage of all patients. The patient who died before 3-month follow-up is not included in the analysis. At 6-month follow-up, two patients with PD were excluded from further analysis resulting in 8 eligible patients. for response assessment. At 12-month follow-up, 6 patients were eligible for response assessment. After 3 months, 50% of all patients achieved a PR. This was also the median time to response. Patients in PR declined at 6-month follow-up to increase again at 12-month follow-up. At each time mark, about 20% of the patients showed PD or had received new IS—this fraction remained constant over time. Mixed response was only seen in a small number of patients at 3- and 6-month intervals

**Fig. 2:**
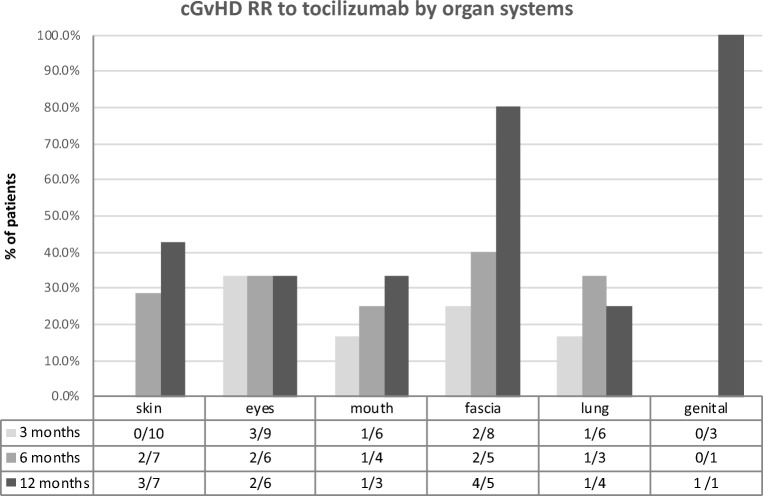
cGvHD response rates to tocilizumab in specific organs. Response of cGvHD per organ: response was assessed by organ grading during tocilizumab therapy at indicated time points. Since not all patients had cGvHD involvement in all organs and some patients received new immunosuppression due to PD, the number of patients at each time point differs (see denominator = *n*). Over the course of 1 year, response rates increased in skin, mouth, fascia, and genital cGvHD. Ocular GvHD RR remained equal over time, while genital involvement improved only in one of three patients after 1 year of therapy

In this single center retrospective analysis on the tolerability and efficacy of tocilizumab as salvage therapy in patients with severe steroid refractory cGvHD, the best overall response rate was 70% (excluding the patient who died before 3-month follow-up, see Table 3). Median time to response was 3 months (5/7 responders), although 2/7 responders only showed significant improvement after 12 months of therapy (Fig. 1). Therefore, unless worsening of cGvHD or severe side effects occurs, continuation of therapy despite SD could be considered, especially in patients with skin and fascial involvement who benefited most from tocilizumab (see Fig. 2). The overall response rate is similar to results of a study in which tocilizumab was used to treat aGvHD as well as cGvHD in steroid refractory patients where the overall response rate was 67% [17]. However, in the latter analyses, only two patients with cGvHD were included—one showed partial response and the other stable disease. In another more recent pediatric study in patients with cGvHD, tocilizumab led to subjective improvement in cGvHD to some degree in all patients, 4/5 patients improved by at least one grade in one organ score, and a reduction of immunosuppression was possible in all patients, even in non-responders [18]. In our study, concomitant immunosuppression with prednisolone was also reduced by 50% in average of all patients at 3- and 6-month follow-up and by another 25% in responders between 6- and 12-month follow-up. In addition, other concomitant IS could be discontinued in 2 of 7 responders (29%). Of note, 5/6 patients failing on ruxolitinib alone responded in combination with tocilizumab, despite known effects on suppression of intracellular IL-6 signaling by JAK-STAT inhibitors. The additive effects may in parts be explained by the fact that ruxolitinib reduces JAK2 (Janus kinase 2) signaling in a quantitative matter while tocilizumab completely abrogates IL-6 effects [22]. In addition, other kinases besides JAK (such as Src and Tec family members) seem to contribute to IL-6 signaling [23]. Two patients in our analysis developed significant infectious complications including sepsis leading to death in one case. The latter patient developed highly elevated C-reactive protein (CRP) and procalcitonin (PCT) levels (with usual septic kinetics) as well as fever although tocilizumab usually suppresses CRP production. Thus, it seemed that the sIL-6R blockade is overruled in situations with very high IL-6 production like severe sepsis. This patient initially suffered from numerous open skin lesions in the context of severe skin cGvHD, increasing the risk for systemic blood stream infection. The other patient (who developed port sepsis) presented with a fever and inadequately low elevation of CRP and PCT levels. However, this patient recovered quickly after antibiotic treatment and port explantation. As these cases demonstrate, clinical risk factors for sepsis should be taken into consideration before initiating tocilizumab treatment since signs and symptoms of sepsis like fever and CRP values can be masked. Hence, patients’ awareness and compliance is pivotal under tocilizumab therapy as they have to contact their treating physician at the earliest signs of infection, and frequent follow-up appointments are obligatory to ensure adequate patient monitoring. Measuring PCT values on a regular basis at follow-ups may help to identify patients developing infections under tocilizumab as PCT values seem unaffected by sIL-6R blockade [24]. At our institution, we provide a 24-h telephone hotline for HSCT patients to ensure immediate contact with a transplant physician experienced in the supervision of patients under immunosuppressive therapy and the peculiarities of tocilizumab.

A recent warning described liver failures after tocilizumab, a side effect not observed in any of our patients. The overall failure-free survival was 55% and the best overall response rate was 70%. Tocilizumab therefore seems a promising treatment option for selected patients with advanced cGvHD but requires further evaluation in phase II clinical trials.
